# Asymptomatic Pancreatic Metastasis from Renal Cell Carcinoma Diagnosed 21 Years after Nephrectomy

**DOI:** 10.1155/2017/8765264

**Published:** 2017-08-30

**Authors:** Megumi Zianne, Naoki Takahashi, Akihiko Tsujibata, Kazuhiro Miwa, Yoshinori Goto, Yutaka Matano

**Affiliations:** ^1^Department of Gastroenterology, Komatsu Municipal Hospital, Ishikawa, Japan; ^2^Department of Gastroenterology, Saitama Cancer Center, Saitama, Japan; ^3^Department of Pathology, Komatsu Municipal Hospital, Ishikawa, Japan

## Abstract

This report presents our experience with a case of pancreatic metastasis of renal cell carcinoma (RCC) at a long-term follow-up after nephrectomy. A 73-year-old man underwent nephrectomy for right RCC 21 years ago; computed tomography (CT) scanning on routine follow-up revealed a solid mass in the tail of the pancreas, and magnetic resonance imaging (MRI) showed some tumors in the head and tail of the pancreas. The patient was asymptomatic and allergic to contrast medium. Therefore we could not perform contrast CT/MRI for further examination to diagnose pancreatic tumors. We undertook endoscopic ultrasonography (EUS) and detected a hypervascular and low echoic mass; tumor tissues were obtained by EUS-guided fine-needle aspiration (EUS-FNA). Pathological diagnosis revealed pancreatic metastasis of clear cell RCC; this was similar to the pathological findings of tumor tissues initially obtained by nephrectomy. EUS-FNA was extremely useful for the definitive diagnosis of a rare type of pancreatic tumor.

## 1. Introduction

Renal cell carcinoma (RCC) is commonly observed in the field of urology. The incidence of newly developed RCC is estimated at 338,000 per year, and estimated cancer deaths due to RCC are 144,000 per year [1]. In Asia, the incidence of RCC is less frequent compared with that in North Europe and South America. Clear cell RCC is one of the most common histological subtypes of RCC that is characterized by malignant epithelial cells with a clear cytoplasm and a nested or acinar growth pattern [2]. Clear cell RCC initially arises from epithelial cells of proximal convoluted tubules of nephrons and invades the renal sinus before extending into the renal vain. Therefore, vascular invasion is more frequently observed in clear cell RCC compared with other histological types of RCC.

In RCC, pancreatic metastasis is less frequent as compared with metastasis to other organs such as lung, bone, and liver [3]. Enhanced computed tomography (CT) and magnetic resonance imaging (MRI) scan are valuable to discriminate between a metastatic tumor arising from RCC and a primary tumor of the pancreas [4–6]. Endoscopic ultrasound-guided fine-needle aspiration (EUS-FNA) is another significant method to confirm pathological diagnosis of pancreatic tumors before surgical treatment. In this study, we report a case with pancreatic metastasis of RCC at a long-term follow-up after nephrectomy.

## 2. Case Report

**Figure 1:**
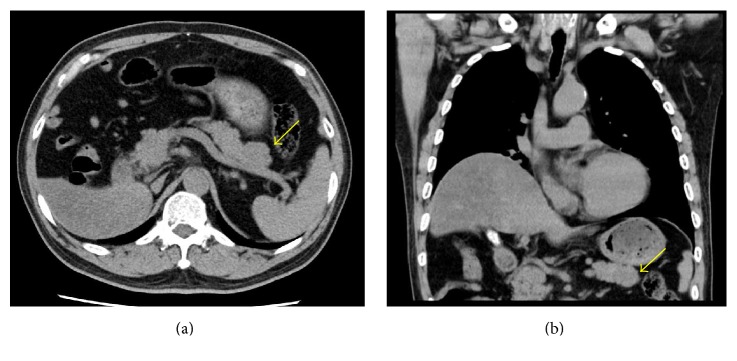
Abdominal computed tomography imaging demonstrating a 27 mm mass in the tail of the pancreas (yellow arrow).

**Figure 2:**
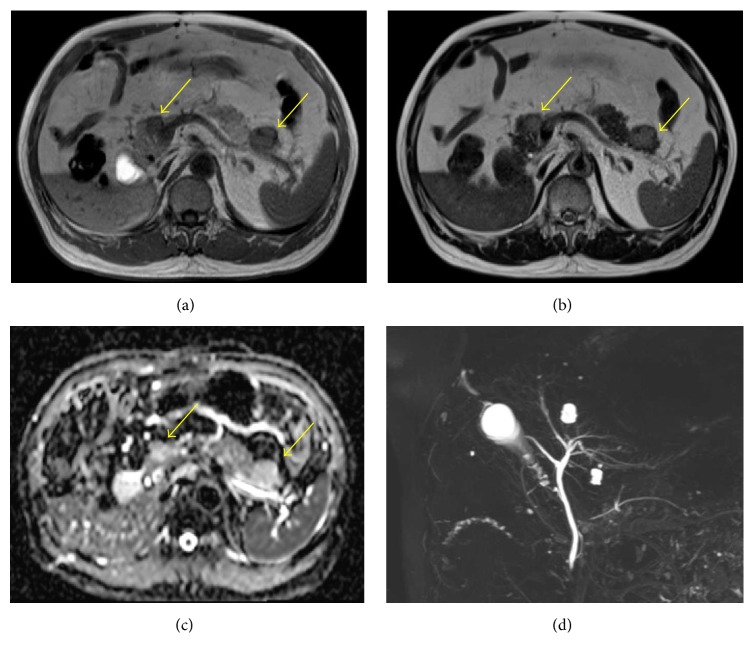
*Magnetic resonance imaging sections of pancreas tumors*. Pancreatic tumors were shown as slightly high-intensity regions on T1-weighted imaging and as isointense regions on T2-weighted imaging (yellow arrow: (a), (b)). Diffusion-weighted imaging (DWI) revealed these masses as high-intensity regions (yellow arrow, (c)), and magnetic resonance cholangiopancreatography (MRCP) showed no stenosis and irregularity in the biliary duct and pancreatic duct (d).

**Figure 3:**
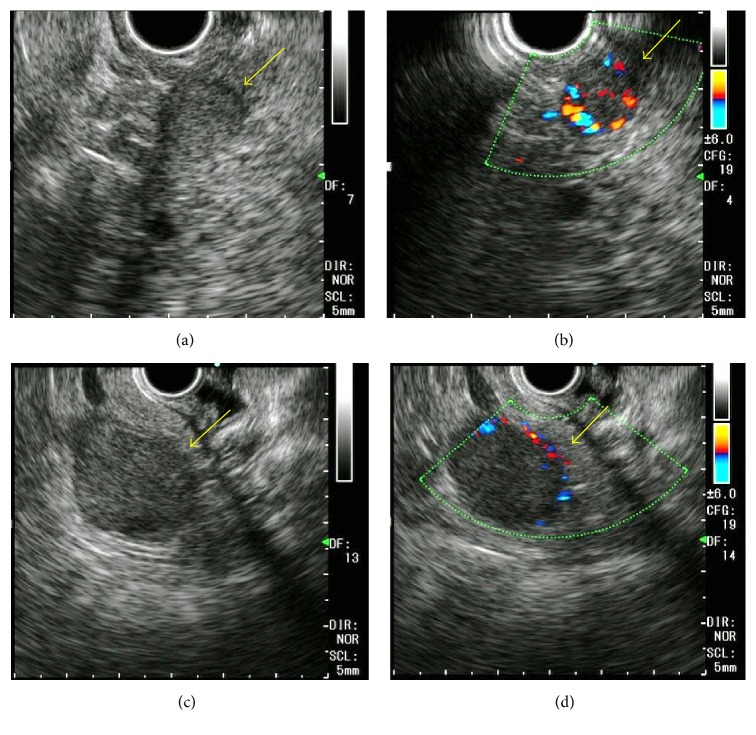
*Endoscopic ultrasonography (EUS) imaging sections of pancreas tumors*. Pancreatic tumors are shown by Convex EUS. A tumor in the head of the pancreas is shown as yellow arrows in (a) and (b) and that in the tail of the pancreas is shown as yellow arrows in (c) and (d). Both tumors had similar findings and hypervascularity in tumor was demonstrated by Doppler mode of EUS.

A 73-year-old man had undergone a right nephrectomy with a retroperitoneal approach for RCC in our hospital 21 years earlier, with a pathological diagnosis of clear cell RCC (intermediate type, INF *β*, G2 > G1, pT3aN0M0, pStage III; UICC 7th edition). Curative resection was confirmed pathologically, and no adjuvant chemotherapy was administered postoperatively. During a routine follow-up assessment by the urology department, a CT scan revealed a slightly low-density mass (27 mm in diameter) in the tail of the pancreas, with no tumor lesions in other organs (Figure 1). Therefore, the patient was referred to the Department of Medical Gastroenterology for intensive evaluation of the pancreatic tumor. At the first visit to our department, the patient was asymptomatic, without abdominal pain or weight loss. In addition, there were no abdominal physical findings except for an operative scar in the right lateral region of the abdomen. Laboratory blood test at the first visit showed that blood urea nitrogen (BUN), creatinine, and amylase in serum were mildly elevated, whereas tumor markers such as carcinoembryonic antigen (CEA), carbohydrate antigen 19-9 (CA19-9), and neuron-specific enolase (NSE) in blood were within the normal range. Contrast-enhanced CT/MRI could not be performed because the patient was allergic to the contrast medium. Noncontrast MRI revealed two lesions: central signals were of slightly higher intensity on T1-weighted imaging but showed isointensity on T2-weighted imaging. The marginal capsule of tumors is shown as low-intensity structures on T1- and T2-weighted imaging. The size of the mass in the head and tail of the pancreas was 12 and 24 mm in diameter, respectively. Diffusion-weighted imaging revealed this mass as having high intensity, and magnetic resonance cholangiopancreatography showed no stenosis or irregularity in the pancreatic duct. These MRI imaging scans are summarized in Figure 2. Convex EUS could detect two lesions, which were a hypoechoic and homogenous mass in the head (12 mm diameter) and tail (22 mm diameter) of the pancreas. Color Doppler EUS revealed that these masses had homogeneous hypervascularity. Findings of EUS are shown in Figure 3.

**Figure 4:**
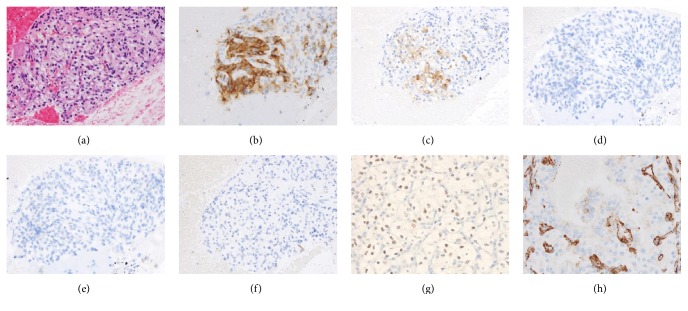
*Pathological findings of pancreatic tumor obtained by EUS-FNA and surgical resection*. Tumor tissues were obtained from a tumor of the tail of the pancreas. Tumor tissue comprised a cluster of mild atypical cells with clear cytoplasm on hematoxylin and eosin staining (a). IHC stain of tumor tissue obtained by EUS-FNA showed that CD10 (b) and NSE (c) were positive and synaptophysin (d), MUC6 (e), and cytokeratin 7 (f) were negative. PAX-8 (g) and vimentin (h) were positive in tumor tissue obtained by surgical resection.

In this case, tumor of pancreas head was not clearly detected by CT scan. EUS is most detected pancreas lesion and EUS-FNA is considered as most safety method as biopsy. We performed EUS-FNA using an EZ shot 22G (Olympus, Tokyo, Japan) to confirm the pathological diagnosis of these masses. Biopsies were performed for each mass, and tumor tissues were gathered from a biopsy specimen of the tail of the pancreas. Tissue smear slides are shown in Figure 4; tumor tissue comprised a cluster of atypical cells with a clear cytoplasm on hematoxylin and eosin (HE) staining, and clear cell RCC was strongly suspected. Immunohistochemical staining was additionally performed to confirm a definitive diagnosis. Immunohistochemistry (IHC) was positive for CD10 and NSE and negative for cytokeratin 7, synaptophysin, and MUC6 (Figure 4). Finally, the pancreatic tumors were pathologically diagnosed as pancreatic metastasis from clear cell RCC.

Curative resection for pancreas lesions was considered as most suitable treatment. On the other hand, surgical resection was very invasive and elderly patients have more risk of any complications after surgery compared with younger patients. In addition, he had taken drugs for the diabetes mellitus and mild nephropathy. After surgery, control of blood sugar level is considered to be worse. We recommended two choices: (1) surgical resection of pancreatic lesions on the basis of previous reports and (2) molecular targeted therapy as a treatment for a metastatic stage of RCC. The patient selected surgical treatment. The findings of the pathological diagnosis of surgical specimens closely resembled those of the diagnosis of clear cell RCC using EUS-FNA. PAX-8 and vimentin were positive in tumor tissues obtained through surgical resection (Figure 4).

## 3. Discussions

We reported a case of metastatic tumors of the pancreas arising from RCC at a long-term follow-up after curative nephrectomy. Previous case reports of pancreatic metastasis of RCC diagnosed using EUS-FNA after curative nephrectomy exist [7–16]. Owing to the recent development of EUS-FNA, we could obtain a pathological diagnosis by less invasive procedures, without surgical resection, and take an appropriately planned treatment decision. As described in our case report, EUS-FNA may be especially effective for diagnosis of a pancreatic tumor posing difficulties for diagnostic imaging because of a contrast-medium allergy.

Recurrence rates after nephrectomy for RCC are 20%–40% and median time to recurrence is 15–18 months, according to previous reports [17]. Conversely, late recurrences more than 10 years after surgery are more frequently observed in RCCs compared with other malignant tumors. According to a previous meta-analysis that evaluated pancreatic resection for malignant tumors, survival time and recurrence-free interval after pancreatic resection in RCC were significantly longer than that in other types of tumors [18]. Previous reports indicated that pathological features of most cases with pancreatic metastasis of RCC were a low-grade tumor, and patients with a high-grade tumor were associated with a short survival time [5]. This finding supports the mechanism of late recurrence after nephrectomy for RCC, and the nuclear grade of tumor cells in the present case was also low. Main symptoms at the time of initial recurrence were reported as pain, jaundice, and bleeding in a previous report. Conversely, pancreatic metastasis of RCC is detected without any symptoms in up to 50% of cases [19]. In the present case, there were no symptoms and the pancreatic tumor was incidentally detected by a CT scan 21 years after nephrectomy. Generally, the follow-up period after curative resection for advanced solid tumors is 5 years; however, longer follow-up may be required for patients with high risk of RCC recurrence. Unfortunately, adjuvant treatment after nephrectomy for RCC is controversial, as indicated by a recent phase 3 randomized trial [20, 21]. Therefore, evidences for an appropriate follow-up period after nephrectomy for RCC have not been established by prospective studies.

Metastatic and primary pancreatic tumors need to be differentiated when a pancreatic tumor mass is detected. Pancreatic ductal carcinoma is most frequently observed in solid pancreatic tumors and is usually detected as a hypovascular tumor on a contrast CT scan. On the other hand, the solid pseudopapillary neoplasm, serous cystic neoplasm (solid type), acinar cell carcinoma, endocrine tumor, and metastatic tumor of the pancreas are detected as hypovascular tumors. Frequencies of these hypervascular tumors are rare, and pathological diagnosis is required because treatment strategies differ based on tumor histology. EUS-FNA is effective in providing an accurate diagnosis identifying surgical candidates and avoiding potentially unnecessary surgery.

As treatment for patients with recurrent RCC, surgical resection or molecular targeted therapy is often reported. In an evaluation of the clinical benefit of surgery for pancreatic metastasis from RCC, Tanis et al. gathered individual data of pancreatic metastasis of RCC from a published series and compared survival time between the surgical and nonsurgical groups [22]. The 5-year survival rate was 72.6% in 311 resection patients and 14% in 73 nonresection patients. In addition, extrapancreatic disease is an independent risk factor for recurrence after pancreatic resection. Zerbi et al., moreover, reported that the 5-year survival rate was 88% in 23 resection patients and 47% in 13 nonresection patients in RCC with pancreatic metastasis [23]. All patients who could undergo surgical resection comprised a favorable risk group of MSKCC. Owing to the development of molecular targeted therapy, survival time of the nonsurgical group may have recently improved compared with that previously reported.

Molecular targeted drugs markedly improved survival times of RCC patients in the recent decade [24–28]. Furthermore, recent development of immune checkpoint inhibitors provides a new treatment strategy in malignant solid tumors. Among patients previously treated for advanced RCC, nivolumab, a programmed death-1 inhibitor, significantly improved the response rate and overall survival compared with everolimus in a randomized phase 3 trial [29]. If patients with physical complications are considered to be intolerant of surgical treatment, these molecular drugs and immune checkpoint inhibitors may be the first choice of treatment. Because the frequency of severe adverse events of immune checkpoint is well known to be quite lower than that of molecular target inhibitors, even elderly patients can be treated safely by a PD-1 inhibitor.

Immunohistochemical evaluation for the metastatic tumor was effective for the diagnosis of primary tumor. PAX-8 is commonly expressed in epithelial tumors of the thyroid and parathyroid glands, kidney, thymus, and female genital tract [30]. In our study, CT scan showed no tumors in the thyroid gland, parathyroid glands, and thymus. PAX-8 expression strongly indicated the possibility of pancreatic metastasis from RCC. In addition, a previous report indicated that CD10 positivity is useful for pathological diagnosis of clear cell or papillary RCC, and we could eliminate the chromophobe RCC [31]. Pathological finding of clear cell RCC by HE staining is a characteristic feature, and the addition of immunohistochemical evaluation such as PAX-2 or PAX-8, CD10, cytokeratin, vimentin, and epithelial membrane antigen contributes to the definitive diagnosis of a primary lesion in pancreatic metastasis [32].

In conclusion, we report our clinical experience with a case of pancreatic tumor diagnosed by EUS-FNA. Despite a long interval after nephrectomy for RCC, it is necessary to assess the recurrence of RCC. Evidence for an appropriate follow-up period after nephrectomy is not established, and prospective studies evaluating this aspect are required in future. In patients allergic to contrast medium, the vascularity of pancreatic tumors cannot be evaluated by only CT/MRI scan. In such cases, EUS could evaluate the vascularity of tumors and simultaneously obtain tumor tissue samples by EUS-FNA, which is useful for the pathological diagnosis and treatment planning in rare types of pancreatic tumors.
